# Antisense oligonucleotide treatment produces a type I interferon response that protects against diet-induced obesity

**DOI:** 10.1016/j.molmet.2020.01.010

**Published:** 2020-01-30

**Authors:** Kristin M. McCabe, Joanne Hsieh, David G. Thomas, Matthew M. Molusky, Liana Tascau, Jun B. Feranil, Li Qiang, Anthony W. Ferrante, Alan R. Tall

**Affiliations:** 1 Division of Molecular Medicine, Department of Medicine, Columbia University, New York, NY, 10032, USA; 2 Naomi Berrie Diabetes Center, Department of Medicine, Columbia University, New York, NY, 10032, USA; 3 Naomi Berrie Diabetes Center, Department of Pathology and Cell Biology, Columbia University, New York, NY, USA

**Keywords:** Obesity, White adipose tissue, Type I interferon, Antisense oligonucleotides, Adipose tissue macrophages

## Abstract

**Objective:**

In mouse models, deficiency of TTC39B (T39) decreases hepatic lipogenic gene expression and protects against diet-induced steatohepatitis. While assessing the therapeutic potential of antisense oligonucleotides (ASOs) targeting T39, we discovered an unexpected weight loss phenotype. The objective of this study was to determine the mechanism of the resistance to diet-induced obesity.

**Methods:**

To assess therapeutic potential, we used antisense oligonucleotides (ASO) to knock down T39 expression in a Western or high-fat, high-cholesterol, high-sucrose-diet-fed *Ldlr*^*−/−*^ or wild-type mice.

**Results:**

T39 ASO treatment led to decreased hepatic lipogenic gene expression and decreased hepatic triglycerides. Unexpectedly, T39 ASO treatment protected against diet-induced obesity. The reduced weight gain was seen with two different ASOs that decreased T39 mRNA in adipose tissue macrophages (ATMs), but not with a liver-targeted GalNac-ASO. Mice treated with the T39 ASO displayed increased browning of gonadal white adipose tissue (gWAT) and evidence of increased lipolysis. However, T39 knockout mice displayed a similar weight loss response when treated with T39 ASO, indicating an off-target effect. RNA-seq analysis of gWAT showed a widespread increase in type I interferon (IFN)-responsive genes, and knockout of the IFN receptor abolished the weight loss phenotype induced by the T39 ASO. Some human T39 ASOs and ASOs with different modifications targeting *LDLR* also induced a type I IFN response in THP1 macrophages.

**Conclusion:**

Our data suggest that extrahepatic targeting of T39 by ASOs in ATMs produced an off-target type 1 IFN response, leading to activation of lipolysis, browning of WAT, and weight loss. While our findings suggest that ASOs may induce off-target type 1 IFN response more commonly than previously thought, they also suggest that therapeutic induction of type 1 IFN selectively in ATMs could potentially represent a novel approach to the treatment of obesity.

## Highlights

• T39 ASOs produced an off-target type I IFN response resulting in reduced obesity, increased lipolysis and browning of WAT.
• The resistance to diet-induced obesity and browning of white adipose tissue was dependent on IFNAR1.
• Multiple ASOs produced a type I IFN response in vitro in human THP1 cells, suggesting they may be more common than suspected.
• Therapeutic induction of low-grade type 1 IFN responses by ASO targeting to ATMs may represent a novel treatment for obesity.

## 1 Introduction

Obesity is a major risk factor for the development of metabolic diseases, including type 2 diabetes, cardiovascular disease, and non-alcoholic fatty liver disease (NAFLD) [1]. The increasing rates of obesity and its complications have led to a search for novel therapeutics to induce weight loss [2]. White adipose tissue (WAT) stores excess energy as lipids that can be released as fatty acids during times of need. Brown adipose tissue (BAT) has the ability to dissipate this energy as heat to maintain body temperature. Brown adipocytes are more metabolically active than white adipocytes, and they have large numbers of mitochondria and express uncoupling protein 1 (*Ucp1*), which uncouples electron transport from adenosine triphosphate (ATP) production, producing heat [3]. Certain stimuli can cause metabolically active adipocytes (called beige or brite adipcytes) to develop within WAT, a process known as beigeing or browning of WAT [4]. Increasing the browning of WAT might be a novel approach for the treatment of obesity [[4], [5], [6]]; however, practical ways to achieve this have not yet been brought to the clinic.

Antisense oligonucleotides (ASOs) are an emerging therapeutic platform that have been approved for the treatment of a variety of diseases including spinal muscular atrophy (SMA), hATTR amyloidosis, and familial chylomicronemia syndrome (in the European Union). Antisense therapy works by binding to the complementary mRNA and targeting it for RNase H-mediated degradation. TTC39B (T39) is a scaffolding protein that promotes the ubiquitination and degradation of liver X receptor (LXR), a transcription factor that rids the body of excess cholesterol [7]. T39 deficiency in mouse models resulted in protection from diet-induced steatohepatitis and improved the lipoprotein profile [7]. These findings suggested that T39 might be a novel therapeutic target.

This study was initiated to investigate the use of ASOs to knock down T39 expression, reduce fatty liver, and improve lipoprotein cholesterol levels. Unexpectedly, while T39 ASO treatment reduced fatty liver, it also suppressed adiposity induced by a high-fat diet. Exploring the underlying mechanisms, we discovered an off-target induction of a type 1 interferon (IFN) response in adipose tissue, likely originating in macrophages, and showed that this was responsible for weight loss.

## 2 Methods

### 2.1 Mice and diet

Male and female *Ldlr*^*−/−*^, C57Bl/6 J, *Ifnar1*^*−/−*^, and *Mda5*^*−/−*^ mice were purchased from Jackson Laboratory (Bar Harbor, ME). *Ifnar1*^*−/−*^ mice were bred with C57Bl/6 J mice to produce *Ifnar1*^*+/−*^, which were used as breeders to produce *Ifnar1*^*−/−*^ and *Ifnar*^*+/+*^ littermates for all experiments. The experiments started when mice were 8 weeks of age. The mice were given 50 mg/kg/week (or 5 mg/kg/week for GalNAc conjugated) T39 ASO or control ASO via subcutaneous injections for a total of 4 weeks and then were placed on either a western diet (WTD; 21% milk fat, 0.2% cholesterol, no. 88137) (Harlan Teklad, NJ) or a high-fat, high-sucrose, high-cholesterol diet (HFSC, 35.5% fat, 24% sucrose, 0.15% cholesterol no. D09071704) (Research Diets, NJ) for 2–20 weeks, as indicated. ASO injections were continued on a weekly basis throughout the study. Body weights were monitored weekly. Mice were housed in a specific pathogen-free facility on a 12-hour light:dark cycle. Compatible mice of mixed genotypes and the ASO treatment group were housed in groups of five. All animal protocols were approved by the Institutional Animal Care and Use Committee of Columbia University and were carried out in accordance with the National Institutes of Health Guide for the Care and Use of Laboratory Animals.

### 2.2 Antisense oligonucleotides (ASOs)

For in vivo mouse studies, Ionis pharmaceuticals provided two T39 and one control 2′MOE-modified ASO. One of these sequences was also provided as a GalNAc-conjugated ASO. The 2′MOE-modified ASOs were given as weekly subcutaneous injections of 50 mg/kg/week, and the GalNAc-conjugated ASOs at 5 mg/kg/week. Ionis also provided four T39 and one control human cET-modified ASO for in vitro work in THP1 cells. For the low-density lipoprotein receptor (LDLR) human ASOs, we used Qiagen LNA GapmeRs LG00228326-DDA (326), LG00228328-DDA (328), LG00228306-DDA (306), LG00228311-DDA (311), and control LG00000002-DDA (Qiagen) for in vitro work in THP1 cells.

### 2.3 Lipoprotein analysis

Blood samples were collected by tail bleeding into BD microtainers for serum separation (BD), and serum was separated by centrifugation. To assess very low density lipoprotein (VLDL), low-density lipoprotein-cholesterol (LDL-C), and high-density lipoprotein cholesterol (HDL-C) and triglycerides, we performed KBr density ultracentrifugation using a TLA100 rotor in a Beckman Optima TL Ultracentrifuge. To spin off the VLDL fraction (d < 1.001 mg/mL), 20 μL of serum was layered below 200 μL of density 1.001 mg/mL solution and spun for 5 h at 50,000×*g*. After removing the VLDL fraction, the remaining sample was adjusted to a density of 1.063 mg/mL and a volume of 200 μL and spun for 15 h at 70,000×*g* to spin off the LDL fraction (d < 1.063 mg/mL). After removing the LDL fraction, the remaining sample was adjusted to a density of 1.125 mg/mL and a volume of 200 μL and spun for 8 h at 80,000×*g* to spin off the HDL2 fraction (d < 1.125 mg/mL). After removing the HDL2 fraction, the sample was adjusted to a density of 1.21 mg/mL and 200 μL for a final spin of 15 h at 80,000×*g* for the HDL3 fraction (d < 1.21 mg/mL). The total cholesterol and triglycerides from each fraction was assessed using an enzymatic kit from Wako (Cholesterol E) and Thermo Scientific (Infinity Triglycerides), respectively.

### 2.4 Liver triglycerides and cholesterol

Lipids were extracted from a section of liver at 4 °C overnight in 20 × 2:1 chloroform:methanol solution. An equal volume of phosphate-buffered saline (PBS) was added to the chloroform:methanol and mixed vigorously for 2 min and then spun for 15 min at 4 °C at 3000 rpm in a Sorvall™ Legend™ XTR Centrifuge TX-1000 (Thermo Scientific). The bottom layer containing the lipids in chloroform was extracted, and the chloroform was left to evaporate in the fume hood. The lipids were re-suspended in 1 mL of Isopropanol 10% Triton X-100. Cholesterol and triglyceride assays were performed as just described. Phospholipids were measured using a phospholipid-specific enzymatic assay from Wako (Phospholipids C).

### 2.5 RNA extraction and qRT-PCR

Tissues were lysed in TRIzol reagent (Life technologies) and RNA was isolated using the Direct-zol™ RNA MiniPrep kit (Zymo research). cDNA was synthesized using a Maxima first-strand cDNA synthesis kit (ThermoFisher Scientific). mRNA levels were measured using quantitative real-time polymerase chain reaction (qRT-PCR) on a StepOnePlus Real Time PCR system (Applied Biosystems) using fast SYBR green mastermix (Thermo Scientific) or TaqMan™ Fast Advanced Master Mix (Thermo Scienfific). The data were normalized to *Gapdh, Actin*, and *Cyclophilin* housekeeping genes. All primers are listed in Supplemental Table 1.

### 2.6 Immunohistochemistry

Staining was done on paraffin-embedded sections of subcutaneous adipose tissue. For antigen retrieval, paraffin-embedded sections were incubated in a citrate-based antigen unmasking solution (Vector Laboratories) for 20 min in a pressure cooker. The sections were blocked in 2.5% normal goat serum (Vector Laboratories) for 30 min followed by a peroxidase block in in 3% H_2_O_2_ in methanol for 1 h. The sections were incubated in anti-UCP1 antibody (Abcam; ab10983) at 1:250 overnight at 4 °C and were then incubated in secondary antibody, goat anti-rabbit (Vector Laboratories) for 1 h. The sections were then stained using the NovaRED peroxidase (HRP) substrate kit (Vector Laboratories) and counter-stained using hematoxyline solution (Harris modified, Sigma–Aldrich). All photos were taken at a magnification of 20x. The percentage of positive staining was calculated using Image-Pro Plus Software (Media Cybernetics, USA), and an average of four to five photos from two sections were taken per mouse. The average adipocyte size was calculated from 10 adipocytes/slide x 4–5 slides/mouse. All analyses were performed in a blinded fashion.

### 2.7 Indirect calorimetry

After 4 weeks on the HFSC diet, the mice were transferred to the mouse metabolic phenotyping core at Columbia University and allowed to acclimate for 1 week. The Comprehensive Lab Animal Monitoring System (Oxymax/CLAMS, Columbus Instruments) was used for indirect calorimetry measurements. Each mouse was individually housed in chambers of the Oxymax/CLAMS system and allowed to acclimate in these chambers for 2 days prior to the start of data collection. The data were collected using Oxymax/CLAMS software at 25-minute intervals for 3 consecutive days.

### 2.8 ^3^H triolein lipid absorption

To measure lipid absorption, mice treated with either the control or T39 ASO and 5 weeks of HFSC diet were fasted 6 h then given 3 μCi tritiated triolein (PerkinElmer) in 200 μL olive oil by oral gastric gavage. After 3 h, the mice were euthanized. Tissue ^3^H was determined by liquid scintillation counting. Whole tissue was dissolved in 1 mL soluene (Sigma) at room temperature for 24 h. Next, 100 μL H_2_O_2_ was added to BAT to bleach. Hionic-Fluor scintillation cocktail (3 mL, PerkinElmer) was added, and samples were counted on a LS 6500 Multi-Purpose Scintillation Counter (Beckman Coulter).

### 2.9 β3-adrenergic receptor agonist experiment

Mice were treated weekly with subcutaneous injections of either the control or T39 ASO (50 mg/kg/week) and placed on the HFSC diet for 2 weeks. Then, mice were given 0.5 mg/kg CL-316,243 (β3 agonist) daily for 3 consecutive days. Mice were euthanized 1 h after the third dose of CL-316,243.

### 2.10 Protein extraction and western blotting

Tissues were lysed in M-PER™ mammalian protein extraction reagent (ThermoFisher Scientific) supplemented with Halt™ protease inhibitor cocktail (1X, ThermoFisher Scientific), Halt™ phosphatase inhibitor cocktail (1X, ThermoFisher Scientific), calpain inhibitor 1 (50 μM, Cayman chemical), and phenylmethylsulfonyl fluoride (PMSF; 10 μg/mL, Sigma–Aldrich). Proteins were resolved by sodium dodecylsulfate-polyacrylamide gel electrophoresis (SDS-PAGE) and transferred to a polyvinylidene fluoride membrane for western blotting using phosphorylated hormone-sensitive lipase (P-HSL) S563 (cell signaling) and β-actin (sigma). The density value of immunoblot signals was quantified by ImageJ.

### 2.11 RNA-seq

For RNA-seq, RNA was isolated from gWAT as just described. RNA was prepared for RNA-seq as described previously [8]. Briefly, RNA with RIN > 8 was subjected to poly-dT pulldown using magnetic beads (NEB) before preparation for RNA-seq using RNA Ultra kits (NEB). Libraries were sequenced on a NextSeq 500 (Illumina), and reads were aligned to the mm10 transcriptome using HISAT2 [9] after adaptor trimming using cutadapt [10]. Read counts per gene for RefSeq genes were computed using featureCounts [11]. Counts were normalized to reads per kilobase per million (RPKM) and processed for pairwise differential expression analysis of selected conditions using DESeq2 [12] with a false discovery rate (FDR)-adjusted p value cutoff of 0.05. Gene ontology analysis was performed using the PANTHER database [13].

### 2.12 Adipose tissue digestion

To separate mature adipocytes from the stromal vascular fraction (SVF) of gonadal white adipose tissue, depots were dissected and minced in Dulbecco modified Eagle medium (DMEM) with 0.2% bovine serum albumin (BSA) then centrifuged at 500×*g* for 5 min. Minced adipose tissue was then transferred to digestion buffer (DMEM, 0.14 U/mL Liberase TM, 20 mg/mL BSA, 50 U/mL DNase I) and incubated in a 37 °C oscillating incubator for 30 min. Digestion was stopped with ethylenediamine tetraacetic acid (EDTA; 5 mM). Samples were then passed through 100-μM filters and centrifuged for 5 min at 500×*g*. The floating layer of adipocytes and the SVF pellet at the bottom were collected separately and homogenized in TRIzol reagent (Life technologies) for RNA isolation as just described or the SVF was collected for flow cytometry or culturing preadipocytes as described in the supplemental material.

### 2.13 THP1 cells

THP1 monocytes cells were purchased from ATCC and maintained in Roswell Park Memorial Institute (RPMI) media supplemented with 10% fetal bovine serum (FBS), 1% PenStrep, and 3.4 μL/L β-mercaptoethanol. Cells were plated at 7 × 10^5^ cells/mL in 24-well plates and treated with 100 nmol/L Phorbol myristate acetate for 72 h to differentiate into macrophages. Then, cells were transfected with 100 μM of T39 or control ASO (Ionis pharmaceuticals), T39 ON-TARGETplus SMARTpool siRNA (L-051155-01, Dharmacon) or control pool siRNA (D-001810-10-05, Dharmacon), or Ldlr or control ASO (Qiagen) and 1 μL/well Lipofectamine RNAiMAX (Thermo Fisher) in Optimem media (Gibco). After 24 h, the optimem was aspirated and replaced with RPMI media with 10% FBS. After an additional 24 h, cells were collected in Trizol for RNA isolation. For poly (I:C) experiments, cells were treated with 0, 1, or 2 μg/mL of poly (I:C) (LMW)/LyoVec™ (InvivoGen) for 12 h prior to collection.

### 2.14 Statistical analysis

All data are represented as mean ± standard deviation (SD) or standard error of the mean (SEM), as indicated in the figure legend. For comparison of two datasets, the Student t test with Benjamini-Hochberg multiple testing correction (when needed) was used to determine significance. For comparison of three or more datasets, a one-way analysis of variance (ANOVA) with the Dunnett multiple comparisons test was used to determine significance. When two variables were being analyzed (i.e., in the case of time-course data, β3-agonist experiment, or weight gain and gene expression in WT vs. Ifnar1−/− with control or T39 ASO), a two-way ANOVA with the Sidak post-hoc test was used to determine significance. The threshold of significance was set at p < 0.05. Statistical analyses were performed using GraphPad Prism 8. For RNA-seq, gene expression differences were evaluated by the Wald test after linear model fitting using DESeq2, and genes significant at 5% false discovery rate (FDR) were considered to be differentially expressed.

## 3 Results

### 3.1 TTC39B (T39) antisense therapy reduces hepatic lipogenic gene expression and dietary obesity

**Figure 1:**
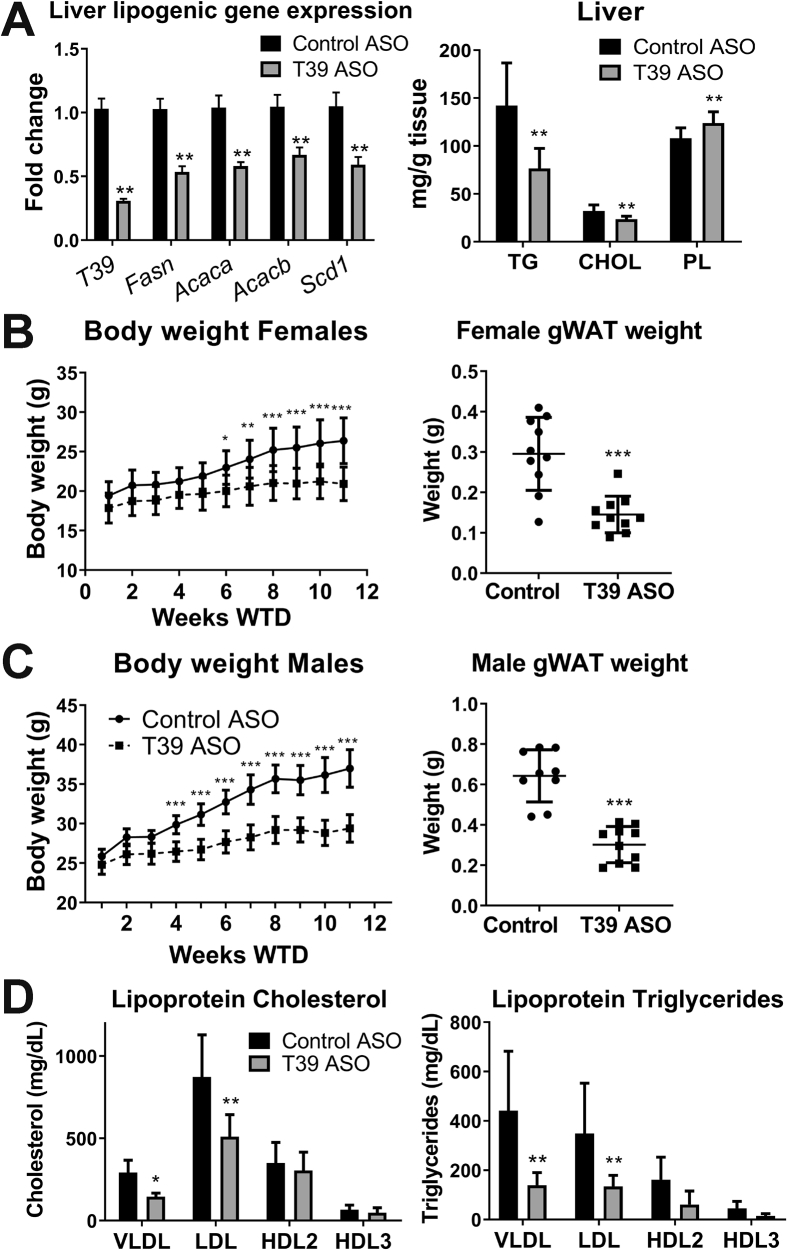
**T39 ASO reduces hepatic lipogenic gene expression and dietary obesity.** Female and male Ldlr−/− mice were treated with a T39 ASO or control ASO and given a western diet (WTD). A) Liver lipogenic gene expression as measured by qRT-PCR and normalized to actin and GAPDH expression and liver triglyceride (TG), cholesterol (CHOL), and phopholipid (PL) content in female mice after 20 weeks WTD. Body weight measured weekly and final gonadal adipose tissue weight (gWAT) in B) female and C) male mice on WTD for 12 weeks. D) Lipoprotein cholesterol and triglycerides in female mice after 20 weeks WTD. Data represent mean ± SEM (A) or SD (B–D), n = 10 mice/group, *p < 0.05, **p < 0.01, ***p < 0.001 compared with control ASO.

To assess the effects on fatty liver, lipoprotein cholesterol, and body weight, WTD-fed *Ldlr*^*/-*^ mice were injected weekly with either T39 or control ASO. After 20 weeks of the WTD, there was a significant decrease in expression of hepatic lipogenic genes including *Fasn, Acaca, Acacb,* and *Scd1* along with a decrease in hepatic triglyceride and cholesterol content (Figure 1A). Unexpectedly, both male and female mice displayed a marked resistance to diet-induced obesity, gaining significantly less weight after 12 weeks of the WTD as well as a decrease in gWAT mass (Figure 1B,C). Along with the reduced body weight, there was a modest improvement in insulin resistance (Supplemental Figs. 1A and B). Similar results were obtained in separate cohorts of *Ldlr*^*−/−*^ or WT mice using a HFSC diet (Supplemental Fig. 1C). T39 ASO-treated female *Ldlr*^*−/−*^ mice had a significant decrease in VLDL and LDL-C and triglyceride levels (Figure 1D), whereas male mice also displayed an increase in HDL-C (data not shown), similar to previous findings in *T39*^*−/−*^ mice [7]. Together, these findings suggested a beneficial metabolic profile resulting from T39 ASO treatment.

**Figure 2:**
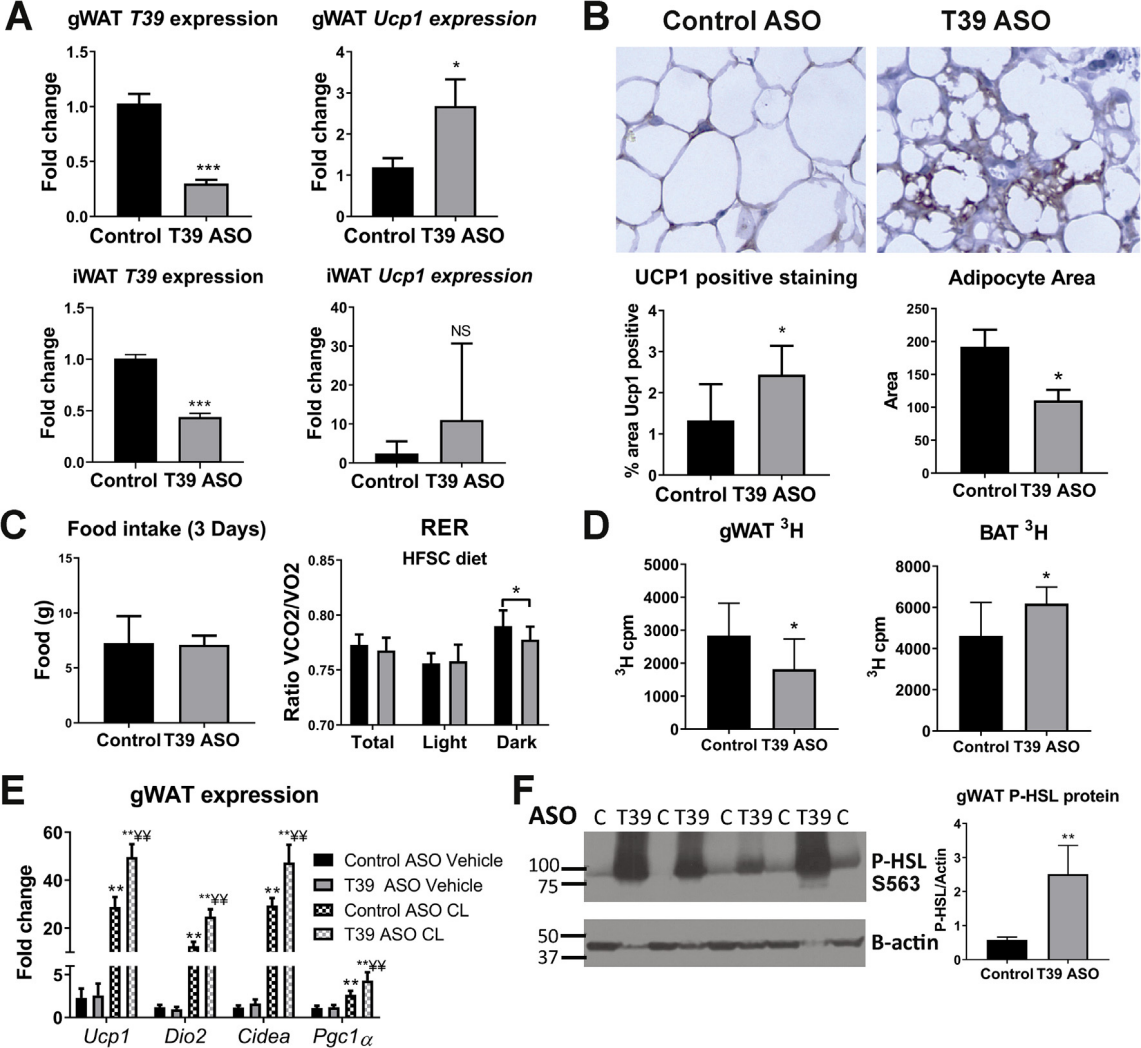
**T39 ASO increases browning of white adipose tissue.** Ldlr−/− or C57Bl/6 J (WT) mice treated with T39 or control ASO and given a WTD (A,B) or HFSC diet (C–F). A) Relative fold change of mRNA expression measured by qRT-PCR in gWAT and iWAT normalized to actin and cyclophilin expression and B) iWAT Immunohistochemistry stained with UCP1 primary antibody in Ldlr−/− mice treated with control or T39 ASO and 12 weeks WTD. C) Total food intake and RER calculated from calorimetry data from WT mice treated with T39 or control ASO and 4 weeks HFSC diet. D) ³H cpm from gWAT and BAT 3 h after oral gavage of ³H triolein in olive oil. E) WT mice treated with T39 or control ASO and 2 weeks of HFSC diet and 3 days of CL-316,243 (CL) or saline injections. Relative fold change of mRNA expression measured by qRT-PCR normalized to actin and cyclophilin expression in gWAT. F) Western blot for P-HSL on tissue lysates from gWAT of WT mice treated with T39 or control ASO and 12 weeks HFSC diet. Graphical representation of P-HSL protein normalized to β-actin level. Data represent mean ± SEM (A,B,E,F) or SD (C,D). n = 8–10/group, *p < 0.05, **p < 0.01, ***p < 0.001 vs. control ASO, ¥p < 0.05, ¥¥p < 0.01 vs. control ASO+β3 agonist.

We conducted further studies to identify the relevant tissue and cell type responsible for reduced adiposity in the T39 ASO-treated mice. The T39 ASO suppressed T39 mRNA levels in both liver and adipose tissue (Figure 1, Figure 2A). A second T39 ASO targeting a different part of the T39 mRNA sequence also caused a decrease in body weight gain on the HFSC diet (Supplemental Fig. 1D). However, using a GalNAc-conjugated T39 ASO, which targets hepatocytes specifically (Supplemental Fig. 1E), there was no effect on diet-induced obesity (Supplemental Fig. 1F). Analysis of SVF and adipocyte fractions of adipose tissue showed that T39 expression was limited to the SVF fraction in both CD45+ and pre-adipocyte (CD45- CD31- CD29+ CD34+) cell fractions (Supplemental Fig. 2A). On further separation of the CD45+ fraction, we found that T39 is expressed in macrophages (CD45+ CD64+), classical dendritic cells (CD45+ CD64- CD11c+), and other CD45+ cell fractions (Supplemental Fig. 2B). Mice treated with the T39 ASO after short-term (2 weeks) HFSC diet feeding appeared to show decreased T39 expression specifically in the macrophage fraction (CD45 + CD64+) of the SVF (Supplemental Fig. 2C). These findings suggest that the resistance to weight gain is not hepatocyte dependent and likely not dependent on mature adipocytes, but may be due to a direct effect within ATMs.

### 3.2 T39 ASO increases browning of white adipose tissue

Mice treated with the T39 ASO had a significant increase in expression of *Ucp1* in gWAT and a trend toward increased *Ucp1* in subcutaneous inguinal adipose tissue (iWAT) (Figure 2A). *Ucp1* is a marker of BAT and its expression is induced in WAT in response to cold or sympathetic activation. Immunohistochemistry analysis using an anti-UCP1 antibody also showed more UCP1-positive staining in iWAT, and the adipocyte size was significantly smaller (Figure 2B). There were no changes in BAT *Ucp1* expression (data not shown). The decrease in diet-induced obesity was not associated with changes in food intake (Figure 2C) or in gWAT expression of *de novo* lipogenic genes (data not shown). A tracer study using ^3^H-labeled triolein administered orally showed a decrease in gWAT uptake and an increase in BAT uptake of ^3^H-fatty acid in T39 ASO-treated mice (Figure 2D). Despite the increase in BAT uptake of fatty acids, there was no change in basal body temperature at room temperature or during a cold challenge (Supplemental Fig. 3). To test whether the mice were more susceptible to browning of WAT, a β3-agonist challenge was performed. Mice were dosed weekly with either the T39 or control ASO and placed on the HFSC diet for 2 weeks. They were then subjected to 3 days of 0.5 mg/kg CL-316,243 injections (β3 agonist) or vehicle control to induce WAT browning. Mice that had been given the T39 ASO had a significant increase in browning gene expression in gWAT, including *Ucp1, Cidea, Dio2*, and *Pgc1α* expression, in response to the CL injections (Figure 2E). In a separate group of mice treated with the control or T39 ASO and 12 weeks of HFSC diet, we found that mice treated with the T39 ASO had significantly more P-HSL S563 as measured by western blot (Figure 2F). We also found that mice treated with the T39 ASO had a decrease in respiratory exchange ratio (RER) as determined in metabolic cages, suggesting that more fat is being used as a fuel source (Figure 2C). However, there was no significant increase in total energy expenditure (Supplemental Figs. 4A and B). These findings led us to consider whether there were differences in lipid absorption. However, following gavage, ^3^H triolein absorption showed no difference in serum or intestinal uptake (Supplemental Fig. 4C), and bomb calorimetry showed no difference in total fecal calorie output (Supplemental Fig. 4D). Taken together, our findings suggest that the T39 ASO induced increased gWAT browning, increased lipolysis in gWAT, and possibly increased fatty acid uptake and oxidation in BAT. The failure to detect increased thermogenesis or increased overall energy expenditure suggests a small effect that produced substantially reduced weight gain over time.

### 3.3 T39 ASO suppresses dietary adiposity in *T39*^*−/−*^ mice

**Figure 3:**
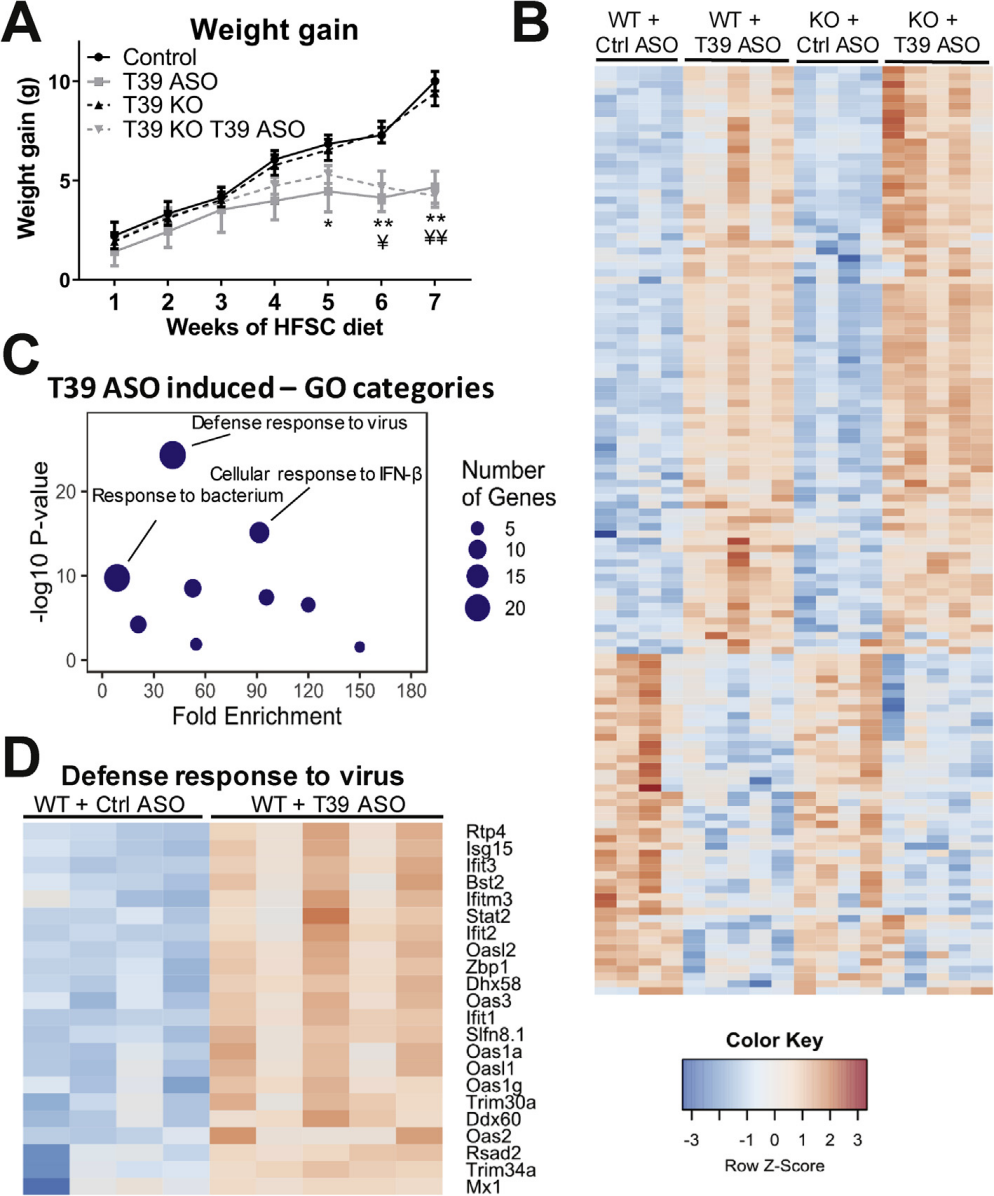
**T39 ASO induces a Type I IFN response in both WT and T39−/− mice.** T39−/− mice and WT littermates were treated with either the control ASO or T39 ASO and placed on the HFSC diet for a total of 10 weeks. A) Weight gain was monitored weekly and B-D) RNAseq was performed on gWAT samples. B) Heatmap of all significant gene changes C) A plot of the GO.

We had not observed consistent changes in body weight in previous studies of *T39*^*−/−*^ mice fed high-fat diets [7], suggesting that the knockout mice might have undergone metabolic adaptation preventing weight loss. Alternatively, the effects of the T39 ASOs could represent an off-target effect. To assess the latter possibility, we examined the effect of the T39 ASO on body weight in *T39*^*−/−*^ mice. This confirmed no difference in body weight increase between *T39*^*−/−*^ and WT mice. Moreover, the T39 ASO produced a similar reduction in body weight in both WT and *T39*^*−/−*^ mice, strongly suggesting an off-target effect of the T39 ASO (Figure 3A, total body weights shown in Supplemental Fig. 5A). To assess whether the effects of T39 ASO on lipogenic genes was an on-target effect, we examined the expression of lipogenic genes in livers from WT or T39 KO mice treated with the control or T39 ASO. These results confirmed that the decrease in lipogenic gene expression was T39 dependent (Supplemental Fig. 6).

### 3.4 RNAseq of gWAT implicates a type I interferon response

To gain further insight into the mechanisms underlying the suppression of dietary obesity by T39 ASO, we performed RNA-seq on gWAT from WT and *T39*^*−/−*^ littermates treated with either the control or T39 ASO. This showed that the T39 ASO was inducing a prominent type 1 IFN response in gWAT of both WT and *T39*^*−/−*^ mice (Figure 3). Fifty-four genes were induced greater than 1.5-fold (Figure 3B), 46 of which (85%) were IFN-inducible. The top two Gene Ontology (GO) categories of induced genes in both WT and *T39*^*−/−*^ mice were ‘defense response to virus’ and ‘cellular response to IFNβ’ (p = 5.0 × 10^−25^, p = 7.6 × 10^−16^ respectively) (Figure 3C,D). In a separate group of mice, we used two different ASO sequences (T39 ASO1 and T39 ASO2) and found that they were both able to induce a type I IFN response in gWAT and caused reduced gWAT weight after 12 weeks of a HFSC diet (Supplemental Figs. 7A and B).

### 3.5 Resistance to diet-induced obesity is IFNAR1 dependent

**Figure 4:**
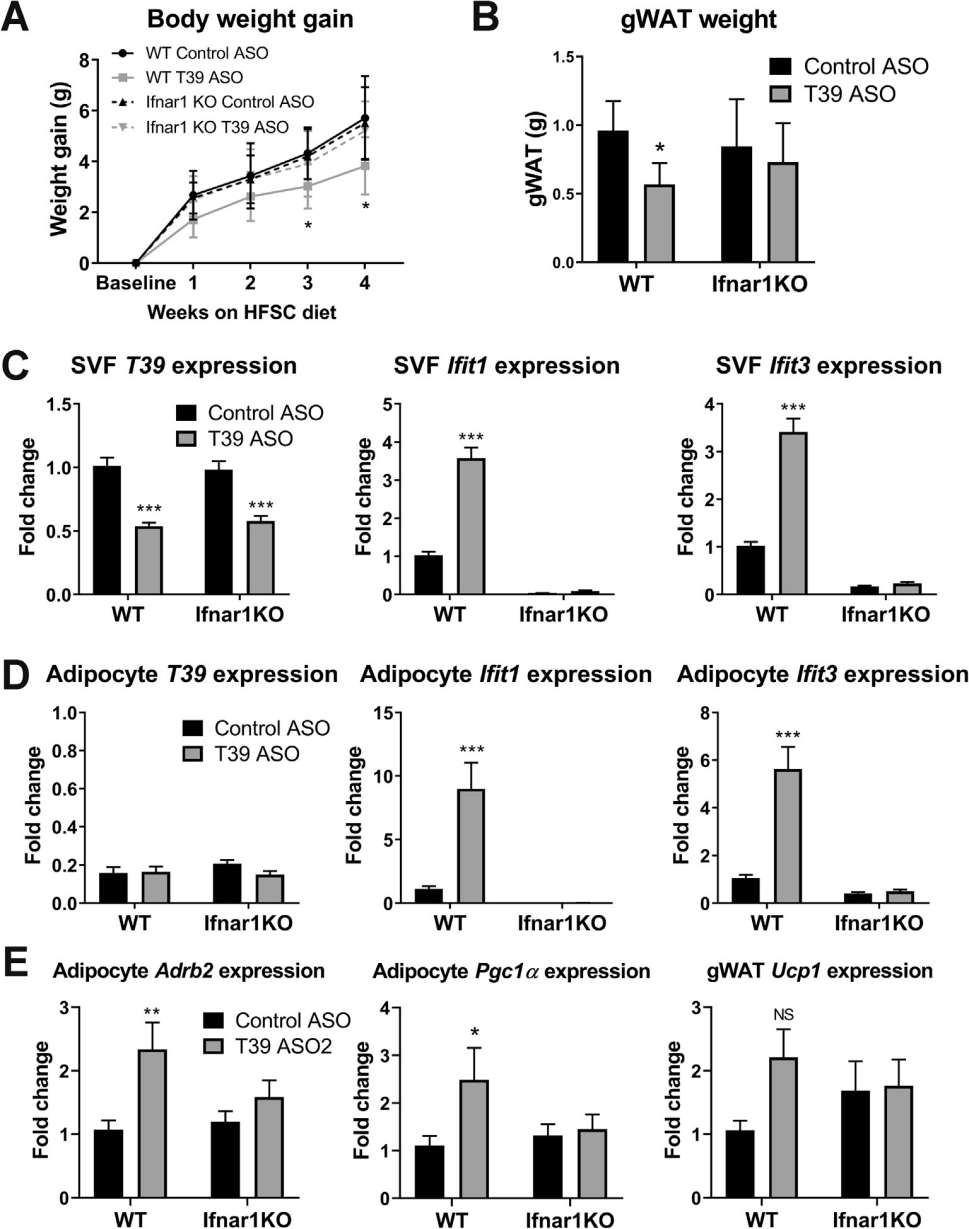
**T39 ASO-induced resistance to diet-induced obesity is IFNAR1 dependent.** Littermate WT and Ifnar1−/− mice were treated with either the control or T39 ASO and placed on an HFSC diet for a total of 5 weeks. A) Body weight gain measured weekly after starting on HFSC diet. B) gWAT weight after 5 weeks HFSC diet. (C–E) Relative fold change of mRNA expression in SVF, adipocytes, or total gWAT measured by qRT-qPCR and normalized to actin and cyclophilin expression. Data represent mean ± SD (A,B) or SEM (C–E), n = 8–11 mice/group, *p < 0.05, **p < 0.01, ***p < 0.001.

To determine if the resistance to diet-induced obesity was dependent on the type I IFN response, *Ifnar1*^*−/−*^ mice that lack subunit 1 of the IFNα/β receptor and its downstream signaling and WT littermate controls were treated with either the control or T39 ASO. The T39 ASO significantly reduced weight gain and gWAT weight in WT mice but not in the *Ifnar1*^*−/−*^ (Figure 4A,B, total body weights are shown in Supplemental Fig. 5B). As expected, the T39 ASO-treated WT mice showed a significant increase in type I IFN genes in both the SVF and adipocyte fractions of gWAT, which was completely absent in the *Ifnar1*^*−/−*^ mice (Figure 4C,D). The increase in browning gene expression seen in the WT mice was also absent in the *Ifnar1*^*−/−*^ mice (Figure 4E), which indicates that the resistance to weight gain and browning of WAT in response to the T39 ASO are both dependent on IFNAR1 and the type I IFN response. There was no detectable circulating IFNβ in T39 ASO-treated mice (<0.94 pg/mL), indicating that this was likely a local response within adipose tissue.

### 3.6 T39 ASO-induced type I IFN response and resistance to diet-induced obesity is not MDA5 dependent

**Figure 5:**
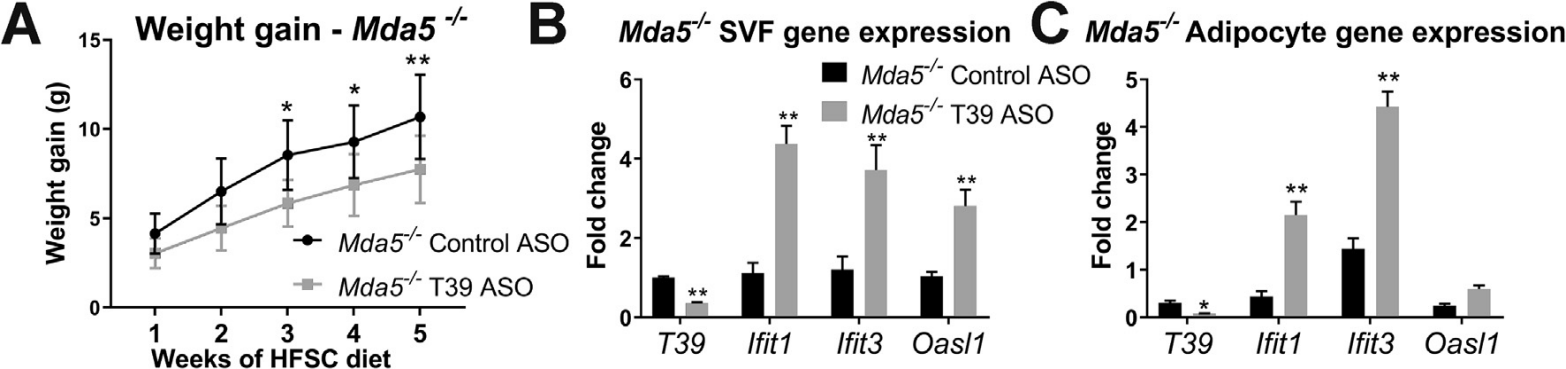
**T39 ASO-induced Type I IFN response and resistance to diet-induced obesity is not MDA5 dependent.** Mda5−/− mice treated with control or T39 ASO and placed on HFSC diet for 5 weeks. A) Body weight gain and B–C) the relative fold change of mRNA expression measured by qRT-PCR normalized to GAPDH and cyclophilin expression in the B) SVF fraction and C) adipocyte fraction of gWAT. n = 7 mice/group, data plotted as mean ± SD (A) or SEM (B–C), *p < 0.05, **p < 0.01 vs. control ASO.

Our discovery of a type 1 IFN response elicited by ASO treatment appears to be relatively novel. A single previous report suggested that a specific 2′MOE-modified ASO could induce a melanoma differentiation-associated protein 5 (MDA5)-dependent off-target type I IFN response [14]. MDA5 is a pattern recognition receptor that recognizes foreign dsRNA and is a sensor for viruses. To test whether this was the mechanism of the observed T39 ASO-induced type I IFN response shown here, *Mda5*^*−/−*^ mice were treated with either the control or T39 ASO and given the HFSC diet for 5 weeks. The *Mda5*^*−/−*^ mice were still resistant to diet-induced weight gain and had increased type I IFN gene expression in gWAT (Figure 5A–C total body weights shown in Supplemental Fig. 5C), suggesting that this effect is independent of MDA5.

### 3.7 The type I IFN response likely originates in macrophages

To further determine if this was a cell intrinsic effect to macrophages, we cultured mouse BMDMs and transfected them with either the T39 or control ASO and found that in differentiated BMDMs, the T39 ASO induced type I IFN genes (Supplemental Fig. 8A). As a comparison, we isolated and cultured pre-adipocytes from gWAT in C57Bl/6 J mice in vitro, and transfected them with either the T39 or control ASO. In primary adipocytes, the T39 ASO did not induce type I IFN genes (Supplemental Fig. 8B). Taken together, this evidence further supports that this effect originates in macrophages.

### 3.8 ASOs targeting human mRNAs can also induce a type I IFN response in cultured THP1 cells

**Figure 6:**
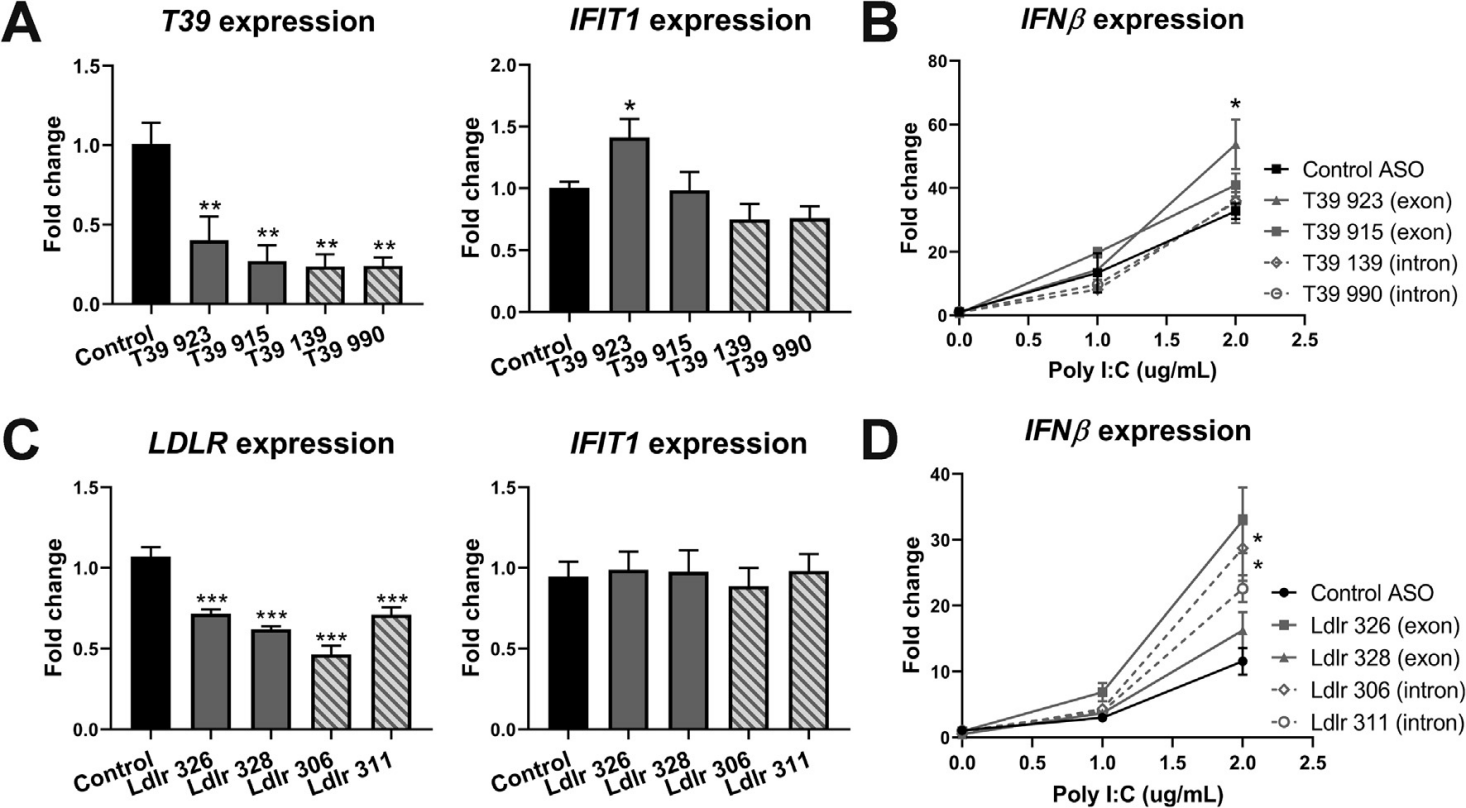
**ASOs targeting human mRNAs can induce a type I IFN response in cultured THP1 cells**. THP1 cells were differentiated into macrophages with 100 nM PMA for 3 days then transfected with ASO for 2 days and collected immediately in trizol for RNA extraction (A,C) or given poly I:C at the indicated doses for 12 h before collection (B,D). Data are the relative fold change of mRNA expression normalized to actin and cyclophilin expression treated with A,B) control or T39 ASO 923, 915, 139, or 990 or C,D) control, Ldlr ASO 326, 328, 306, or 311. B,D) The relative fold change of IFNβ expression after 12 h poly I:C dose response. n = 3 experiments/group (each experiment with 3–4 replicates), data plotted as mean ± SEM, *p < 0.05, **p < 0.01, ***p < 0.001.

Since the reduced dietary adiposity induced by the T39 ASO was likely caused by release of type 1 IFN by macrophages, we performed additional studies to address the specificity of this response using cultured macrophages. The mouse ASOs target exonic sequences, raising the possibility that they were being recognized as foreign DNA in the cytoplasm, for example, as result of formation of DNA-RNA hybrids. To assess the human relevance of these responses, we selected four different ASOs targeting different parts of the human T39 primary transcript including both intronic and exonic sequences and assessed the type 1 IFN responses in human THP1 macrophages. These human ASOs were cET-containing gapmers as opposed to the mouse ASOs, which were MOE-modified gapmers. All ASOs were efficacious at reducing the T39 mRNA. One of the two ASOs targeting exonic sequences produced a type 1 IFN response (ASO 923), whereas none of the ASOs targeting intronic sequences produced a similar response (Figure 6A). In response to increasing doses of poly I:C, the same ASO sequence (923) also produced increased *IFNβ* expression (Figure 6B). We also compared this response with siRNA knockdown of *T39* expression in THP1 cells and found this did not induce a type I IFN response, further supporting that this is an off-target effect of the ASO (Supplemental Fig. 8C). To determine whether the type I IFN response could occur with ASOs designed to targets other than T39, we used Qiagen antisense LNA GapmeRs to knock down *LDLR* expression in macrophages. We designed two ASOs that target exonic regions and two that target intronic regions of *LDLR.* There was no basal induction of a type I IFN response with any of the *LDLR* ASOs; however, two ASOs, one exonic and one intronic, were both able to significantly increase the induction of *IFNβ* expression in response to poly I:C (Figure 6C,D). Together these results showed that a variety of experimental ASOs, with different modifications and targeted to exons or introns, were able to induce an off-target increase in type 1 IFN responses in macrophages.

## 4 Discussion

**Figure 7:**
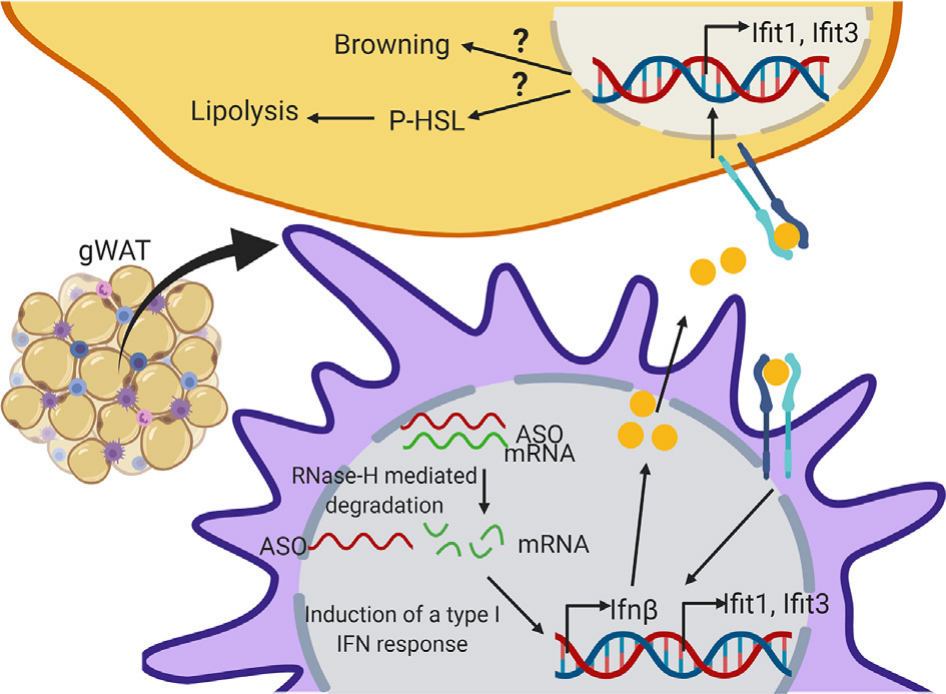
**Summary diagram.** T39 ASO and mRNA target form a double-stranded sequence that is recognized and degraded by RNase-H-mediated degradation. Our data suggest that some product of this triggers the induction of an anti-viral type I IFN response, which induces *Ifnβ* expression. IFNβ is released from the cell and can act in an autocrine or paracrine manner by binding to IFNAR1/2 and inducing the expression of IFN-stimulated genes (ISGs) such as *Ifit1* and *Ifit3*. Within the adipocyte, this can trigger browning of white adipose tissue and lipolysis via still unknown mechanisms. This figure was created with BioRender.com.

We found that treatment with two different T39 ASOs in mouse models of dietary obesity induced browning of gWAT and resistance to diet-induced obesity. T39 is primarily expressed in the SVF of adipose tissue, and the ASO appeared to knock down T39 expression specifically in macrophages, causing an induction of type 1 IFN-responsive genes in both the SVF fraction and in adipocytes. Our data suggest that macrophages can induce type I IFN genes in response to ASO treatment. This response is IFNAR1 dependent; therefore, it is likely that secreted IFN binds to the IFN receptor in an autocrine or paracrine fashion, triggering a type I IFN response in surrounding cells, including adipocytes. This results in an increase in lipolysis, browning of adipose tissue, and resistance to diet-induced obesity (summarized in Figure 7). Since the same type 1 IFN response occurred in WT and T39−/− mice and since some other ASOs and an siRNA effectively targeting T39 did not induce an IFN response in macrophages, we conclude that this response is off-target. Although the magnitude of the effect of T39 ASOs on type 1 IFN responses was modest, it was sufficient to substantially limit dietary obesity. Our findings raise the possibility that therapeutic induction of a persistent low-grade type 1 IFN response in ATMs could represent a potential novel approach to the prevention of obesity.

A major finding from this study was that a T39 ASO-induced type I IFN response in gWAT was able to induce browning of WAT and resistance to diet-induced obesity. RNA-seq analysis of adipose tissue uncovered a clear signature of a type 1 IFN response, and the key role of this response was shown by the lack of an effect of the T39 ASO in mice lacking the IFNAR1. Wieser et al. reported that adipocyte-specific *Ifnar1* knockout mice gained significantly more weight on a high-fat diet with an increase in gWAT mass [15], suggesting a role for a type 1 IFN adipocyte response in the regulation of adiposity. It has long been known that IFNβ [16], IFNα [17], and low-dose LPS [18] can stimulate lipolysis in cultured adipocytes, but the in vivo functional consequences of this have not been previously shown. Our findings suggest that T39 ASO treatment led to an increase in P-HSL, indicating increased lipolysis in adipose tissue. The released fatty acid might then undergo oxidation in BAT, as suggested previously [19]. This also raises a question as to the role of such type 1 IFN responses in physiology. BAT thermogenesis produces heat and can contribute to the febrile response to pathogens [20,21]. Lipolysis of WAT releases fatty acids during times of need to supply other tissues, including BAT [22]. Shin et al. have shown that WAT lipolysis is essential for maintaining body temperature during fasting [23]. Therefore, we speculate that during infection, the type 1 IFN antiviral response triggers lipolysis in WAT to provide a continuous supply of fatty acids to BAT to maintain thermogenesis and increase body temperature. We were unable to document an increase in thermogenesis or total energy expenditure, possibly due to a modest effect that integrated over time to produce significant reduction in adiposity. A power calculation based on observed weight loss parameters and the energy content of fat indicates that a sample size of 62 mice per group would be required to achieve 80% power to detect a difference in energy expenditure of the predicted magnitude at p < 0.05. Thus, our calorimetry experiments with 10 mice per group were underpowered and uninformative.

We found that treatment of mice with the T39 ASO versus the control ASO (a scrambled sequence with no target in the cell) produced a type I IFN-inducible gene response in gWAT as measured by RNA-seq and confirmed with qRT-PCR. Although there have been reports of acute toxicity produced by ASOs [24,25], we have only been able to find one other report describing a type I IFN response [14]. Type I IFNs include IFNα and IFNβ and are involved in mediating the cellular anti-viral response. RNA or DNA viruses trigger the production of type I IFNs that bind to IFNAR1/2, resulting in induction of a host of IFN-inducible genes (ISGs) responsible for modulating the immune response. Numerous anti-viral sensors in the cell respond to foreign double-stranded RNA (i.e., MDA5, RIG-1) or DNA (cGAS-STING) and elicit a type I IFN response. One possible mechanism is that the ASO, when bound to its mRNA target, elicits such a response. A previous report from Burel et al. suggested this possibility [14]. They found that a rare 2′MOE-modified ASO sequence elicited a very large MDA5-dependent type I IFN response. Our data, however, showed an MDA5-independent type I IFN response, and a similar response was found with both mouse T39 ASOs we tested as well as one human T39 ASO in vitro. In addition, using Qiagen-designed LNA GapmeR *LDLR* ASOs targeted to both intronic and exonic sequences, we found one exonic- and one intronic-targeted *LDLR* sequence that also increased the susceptibility to a type I IFN response induced by poly I:C treatment. This finding would argue that cytosolic binding of ASOs is not necessarily required as it was seen with both intronic- and exonic-targeted sequences. Although the Qiagen ASOs do not have the same modifications as the Ionis ASOs, they both function via RNase-H-mediated degradation. Thus, a second possible mechanism is that a product of RNAse-H-mediated degradation is triggering the type 1 IFN response. This idea may seem inconsistent with the finding that the T39 ASO produced an off-target type I IFN response even in T39^−/−^ mice. However, the T39^−/−^ strain that we used has a deletion in exon 2 of the T39 gene, creating a frameshift. Thus, the cognate mRNA likely undergoes nonsense-mediated decay when a premature termination codon is reached during translation [26]. The T39 ASO used here binds to the 3′UTR of the T39 transcript; therefore, it likely binds to the T39 transcript before it is degraded, consistent with the second mechanism suggested above.

Our findings suggest that when using experimental ASOs off-target type 1 IFN, responses may be more common than previously appreciated. We are not aware of any evidence that human therapeutic ASOs, which are more extensively screened, could produce such responses. We did not observe any IFN response with GalNAc-conjugated ASO sequences, which target hepatocytes specifically. Macrophages are likely of key importance in the type I IFN response. When using ASOs for pre-clinical research involving hepatocyte-specific targets, GalNAc-conjugated ASOs, or other tissue-directed ASOs, would prevent the possibility of an off-target type I IFN response. If macrophages are the intended target, rigorous screening for type I IFN responses using macrophage cell culture models should be applied to select sequences that do not induce a type I IFN response.

In our experiments, there was no detectable circulating IFNβ (<0.94 pg/mL), suggesting that the type I IFN response was local and not systemic. Our findings raise the interesting possibility that specific targeting of ATMs in adipose tissue could produce browning of WAT with beneficial metabolic effects. While this approach might seem challenging, a recent report described an siRNA delivery technique in which siRNA is packaged in yeast cell wall glucan shells, GeRPs, and can be delivered specifically to ATMs [27,28]. Such an approach could theoretically be used to deliver TLR3 ligands specifically to ATMs, inducing a type 1 IFN response with potentially beneficial effects on adiposity and insulin resistance.

As the rates of obesity continue to increase, the idea of turning lipid-laden white adipocytes into more energy-consuming brown adipocytes has been an exciting area of research. Although speculative, our findings suggest that selective induction of the type I IFN response within visceral ATMs could represent a novel approach to browning WAT, activation of lipolysis, and the treatment of obesity.
