# Supplementary material for: Antisense oligonucleotide treatment produces a type I interferon response that protects against diet-induced obesity
Source: Mol Metab. 2020 Jan 30;34:146–56. doi: 10.1016/j.molmet.2020.01.010 (PMC7036698; doi:10.1016/j.molmet.2020.01.010)
Supplement: Multimedia component 1 [file mmc1.pdf]

Supplemental Methods:

Glucose and Insulin tolerance tests: For the glucose tolerance test, mice were fasted overnight then given an intraperitoneal injection of 1 g/kg glucose. Blood glucose was measured using a glucometer (Precision Xtra, Abbott) at 0, 15, 30, 60, and 120 minutes. For the insulin tolerance test mice were fasted for 4 hours then given an intraperitoneal injection of 0.75 U/kg insulin. Blood glucose was measured at 0, 15, 30, and 120 minutes.

SVF flow cytometry and cell sorting. SVF isolated from the gWAT as described in the main methods, was incubated in Fc receptor blocking solution (Biolegend) for 30 min then stained with the following antibodies at a dilution of 1:100 to sort the total preadipocyte population (CD45-, CD31-, CD34+, CD29+)(26, 27) FITC anti-mouse CD45 (Biolegend), PE-Cyanine7 anti-mouse CD31 (eBioscience), Alexa Fluor 647 anti-mouse CD34 (BD Biosciences), and PE anti-mouse CD29 (Biolegend). A separate group of mice was used to isolate different CD45+ cell types. SVF was incubated in Fc receptor block for 30 min then stained with the following antibodies at a dilution of 1:100: Pacific blue anti-mouse CD45 (Biolegend), APC anti-mouse CD64 (Biolegend), PE-Cy7 anti-mouse CD11c (Biolegend). Cells were sorted on a BD Aria II Cell Sorter (Columbia Center for Translational Immunology, Flow Cytometry Core) and collected in Trizol for RNA isolation. Data was analyzed using BD FACS diva softwear.

Acute and chronic cold challenge. Body temperature was measured by a subcutaneously implanted IPTT-300 transponder (DMDS). Mice were given ad libitum access to chow diet and placed in a 4°C room for the time indicated. During chronic cold exposure, body temperature was measured at the same time on each day.

Bomb calorimetry. Feces were collected from mice housed individually in calorimetry cages. Samples were dried at 60°C and ground to a fine powder. Calorie content of each sample was determined using a calorimeter (Parr Instrument) run by the New York Obesity Nutrition Research Center Hormone and Metabolite Core Laboratory.

Bone marrow derived macrophages primary cell culture. For generation of bone marrow derived macrophages (BMDMs), bone marrow was isolated from femurs and tibias of female WT C57Bl/6J mice at 8 weeks of age. Bone marrow was differentiated into macrophages by culture in DMEM supplemented with 10% FBS, 1% PenStrep, and 20% L-cell conditioned medium in tissue culture plates in an incubator set at 37°C and 5% CO_2 for 5 days. Macrophages were then re-plated at $1x10^6$ cells/mL in 24 well plates. After 24 hours (day 6) cells were transfected with100 uM of T39/control ASO (Ionis pharmaceuticals) and 2ul/well Lipofectamine RNAiMAX (Thermo Fisher) in Optimem media (Gibco). After 24 hours, Optimem was aspirated and replaced with DMEM 10% FBS 4% L-cell (day 7). After an additional 24 hours, cells were collected in Trizol for RNA isolation and gene expression analysis (day 8).

Primary preadipocyte cell culture. SVF was isolated from gWAT as described in the main methods section, with slight modifications. Cells were digested in L-15 media containing; 1.5% BSA, 1% Pen-Strep, 1mM $CaCl_2$, 2% Hyaluronidase, 0.5% DNase1 and 0.14U/mL Liberase TM. Digestions were allowed to proceed for 1 hour in a 37°C, oscillating, incubator. Digestions were stopped with DMEM + 10% FBS and passed through a 100um filter, centrifuged at 1500 rpms for 8 minutes, and plated at 50,000 cells per well of a 24-well plate. SVF was cultured in DMEM containing 12% FBS for 3 days. Cells were transfected with 100 uM of T39 or control ASO (Ionis pharmaceuticals) and 1.3 ul/well Lipofectamine RNAiMAX (Thermo Fisher) in Optimem media (Gibco) for 8 hours. After 8 hours, Optimem was aspirated and replaced with DMEM containing 12% FBS. After an additional 48 hours, cells were collected in Trizol for RNA isolation and gene expression analysis.

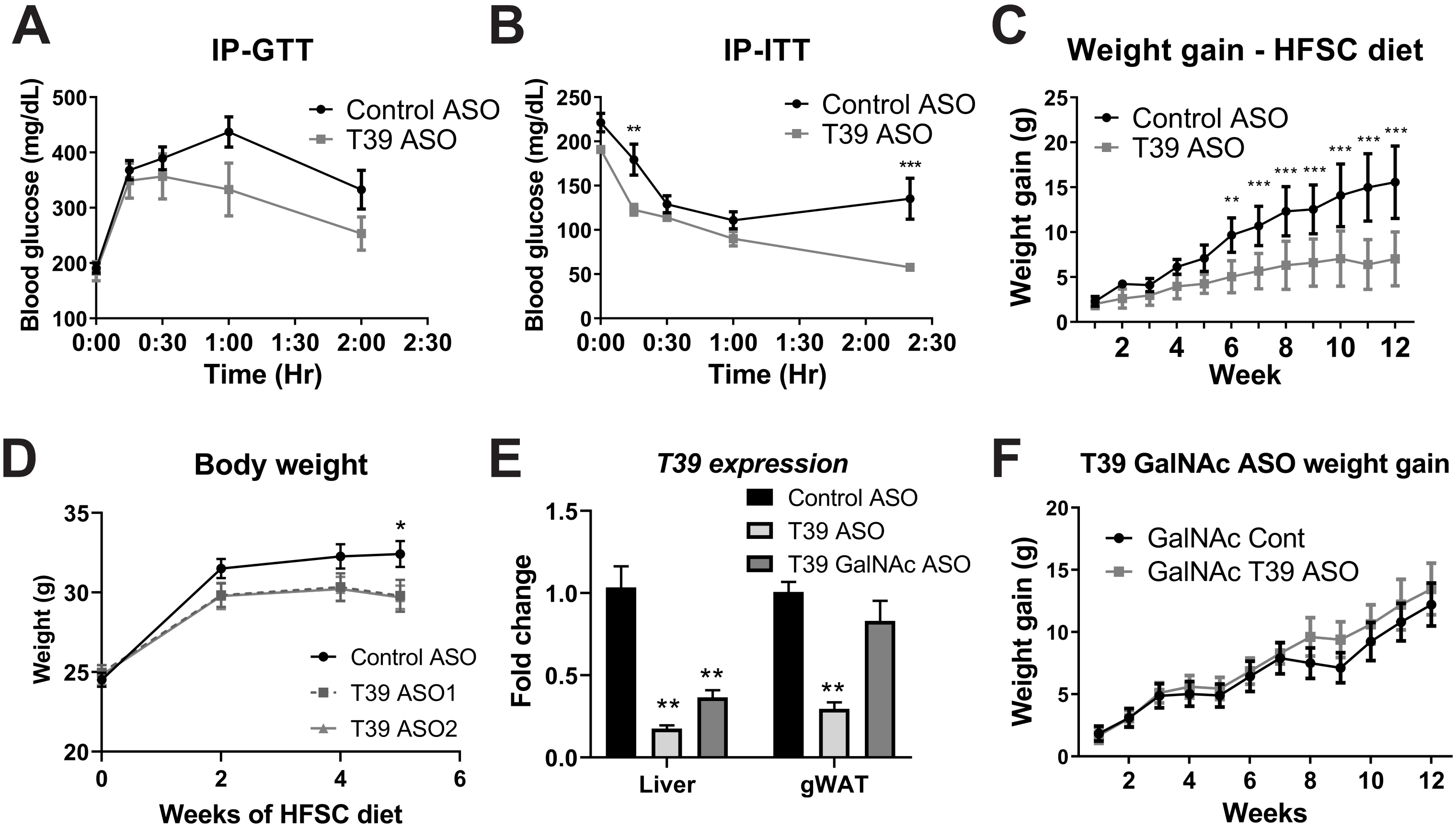

Supplemental figure 1: C57BL/6J mice were treated with either the control or T39 ASO and placed on the HFSC diet. After 9 weeks of diet an A) IP-GTT was performed and then after 10 weeks of diet an B) IP-ITT was performed. C) Body weights were monitored on a weekly basis. D) Body weights of mice treated with either the control ASO, T39 ASO1 or T39 ASO2. E) *T39* expression measured by qPCR and normalized to *Gapdh* and *Cyclophilin* expression in liver and gWAT of mice treated with control, T39, or GalNAc ASO on chow diet. F) Body weights of mice treated with the control or T39 GalNAc conjugated ASO. Data represents mean ± SD, n=10 mice/group *p<0.05, **p<0.01, ***p<0.001.

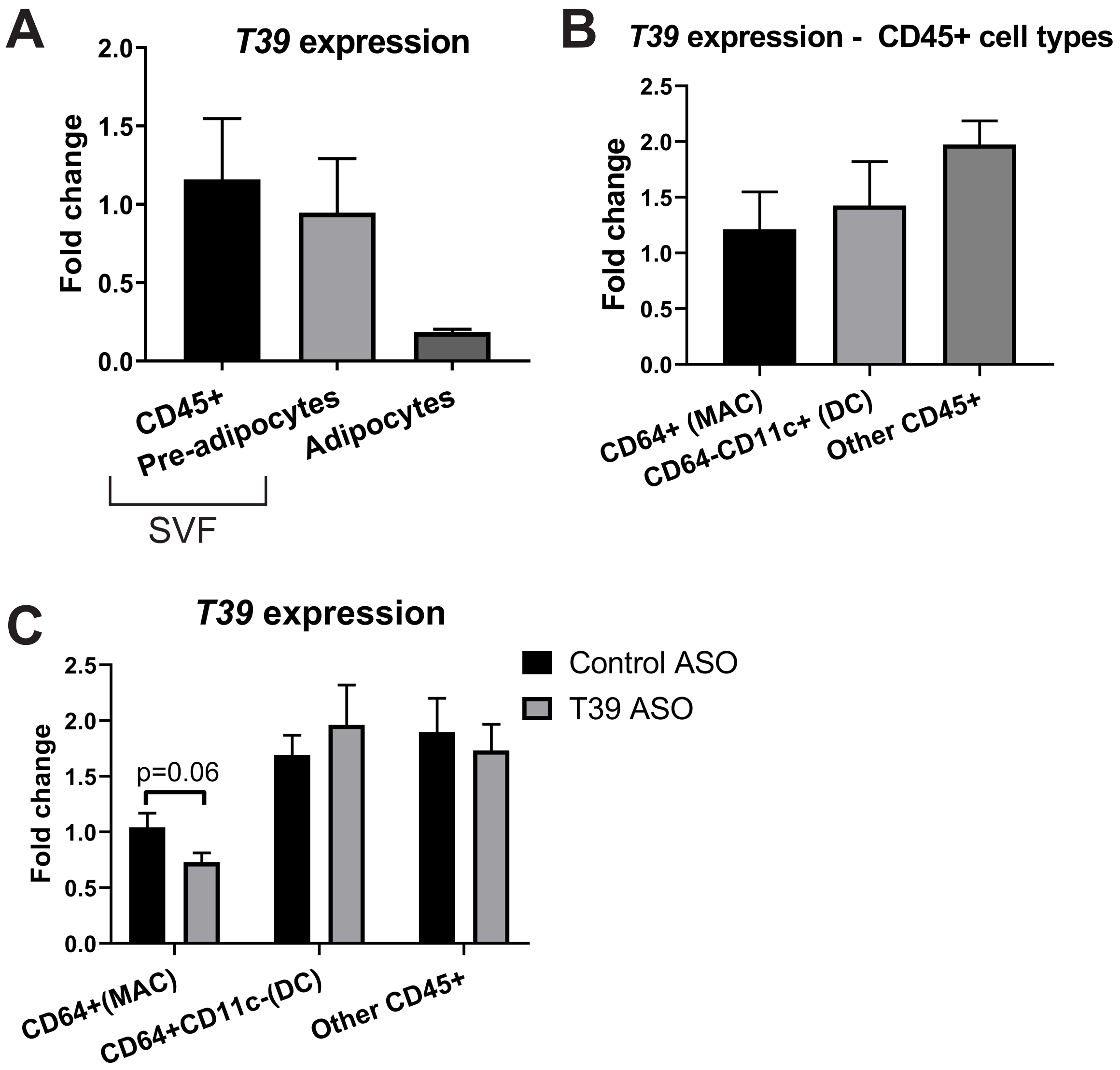

Supplemental Figure 2: A-C) The relative mRNA expression of *T39* normalized to *Cyclophilin* and *Gapdh* from cell populations isolated from gWAT SVF separated from the mature adipocytes in WT C57BL/6J mice on chow diet (A-B) and mice treated with the control or T39 ASO and 2 weeks of HFSC (C). Pre-adipocytes = CD45-CD31-CD29+CD34+ cell population. Data is plotted as the mean ± SEM, n=5-8/group.

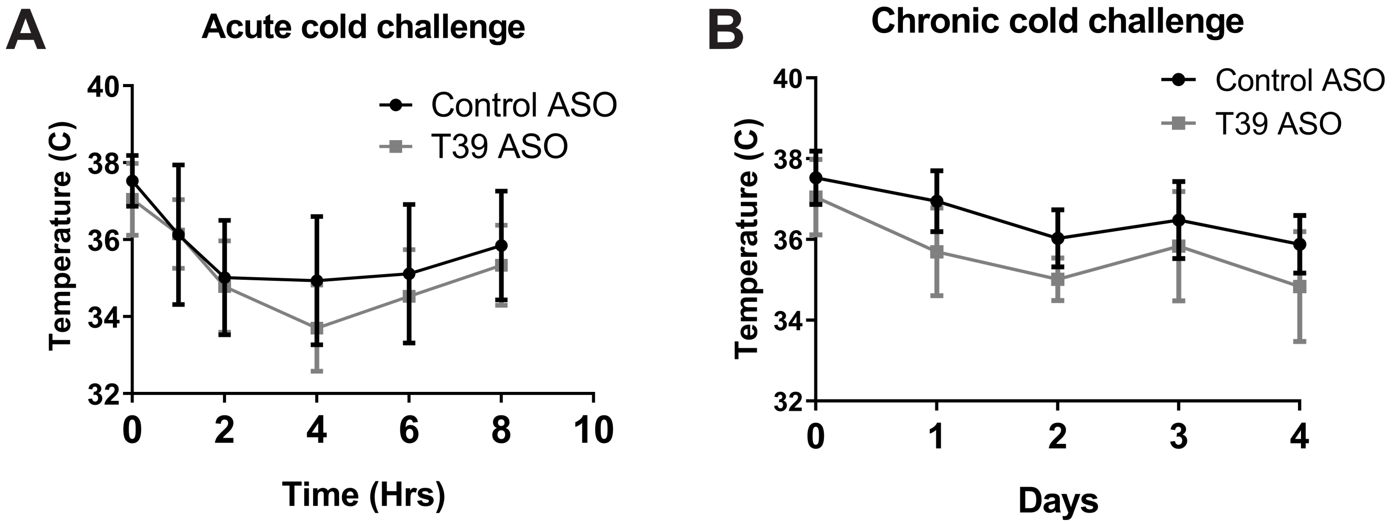

Supplemental figure 3: Core body temperature during acute (A) and chronic (B) cold challenge at 4°C in mice treated weekly with either the control or T39 ASO on chow diet. Time 0 is at room temperature. Data represents mean ± SD, n=6 mice/group.

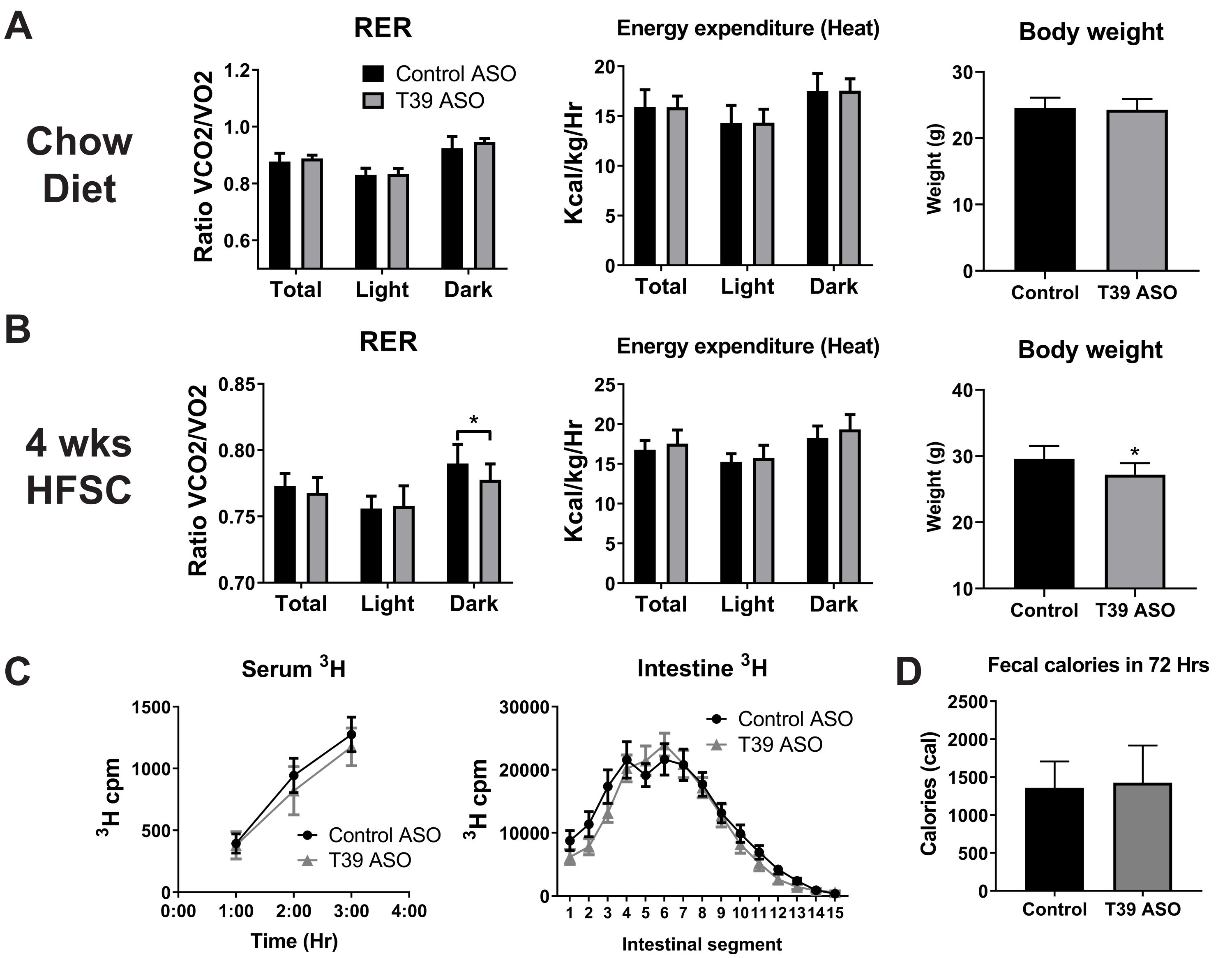

Supplemental figure 4: The respiration exchange ratio (RER), energy expenditure (Heat, kcal/kg/hr), and the body weights (g) from C57Bl6/J mice treated with either the control or T39 ASO in calorimetry cages before (A) and after (B) 4 weeks of HFSC diet. C) ^{3}H cpm measured in serum and the small intestine after an oral dose of 3uCi ^{3}H triolein in mice treated with the control or T39 ASO and 4 weeks of HFSC diet. D) Total fecal calories excreted over a 72 hour period in mice treated with the control or T39 ASO and 4 weeks of HFSC. N=10 mice/group, data represents mean ± SD, *p<0.05.

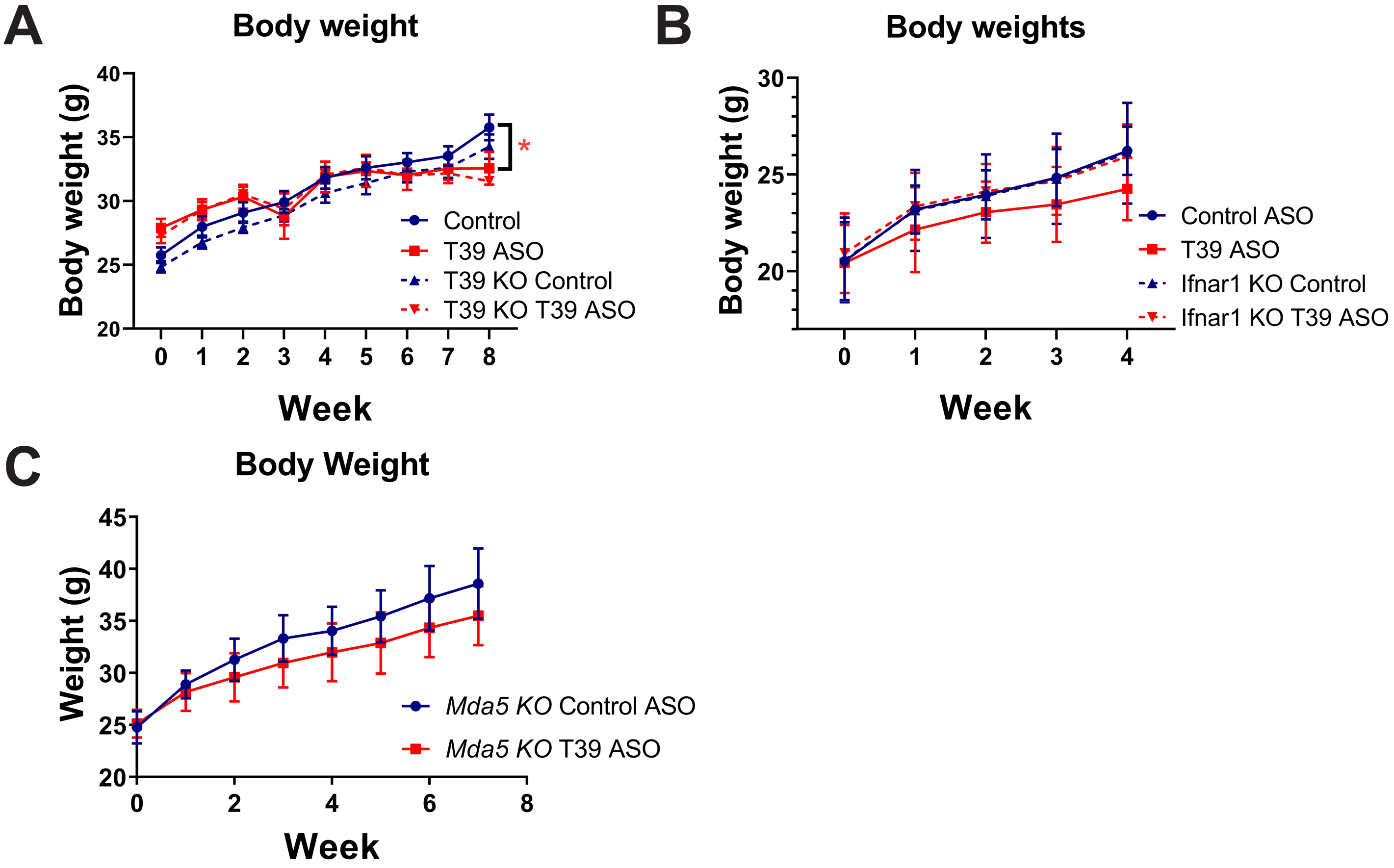

Supplemental figure 5: Raw data of body weight from A) WT or T39 KO littermates treated with either the control or T39 ASO, B) WT or Ifnar1KO littermates treated with either the control or T39 ASO, and C) Mda5 KO mice treated with the control or T39 ASO on HFSC diet. Data represents mean ± SD, n=6-11 mice/group, *p<0.05.

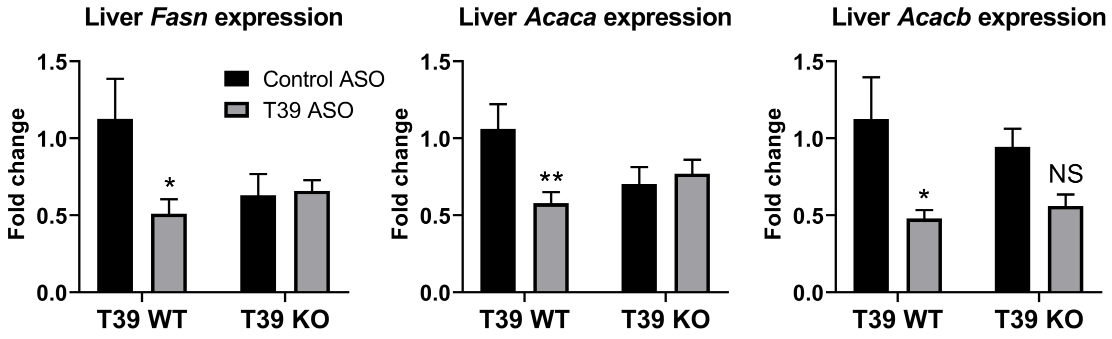

Supplemental Figure 6: Liver gene expression measured by qPCR and normalized to *Actin* and *Gapdh* expression in T39 WT or KO littermates treated with either the control or T39 ASO and placed on HFSC diet for 8 weeks. Data represents mean ± SEM, n=6-8 mice/group *p<0.05, **p<0.01, NS=not significant.

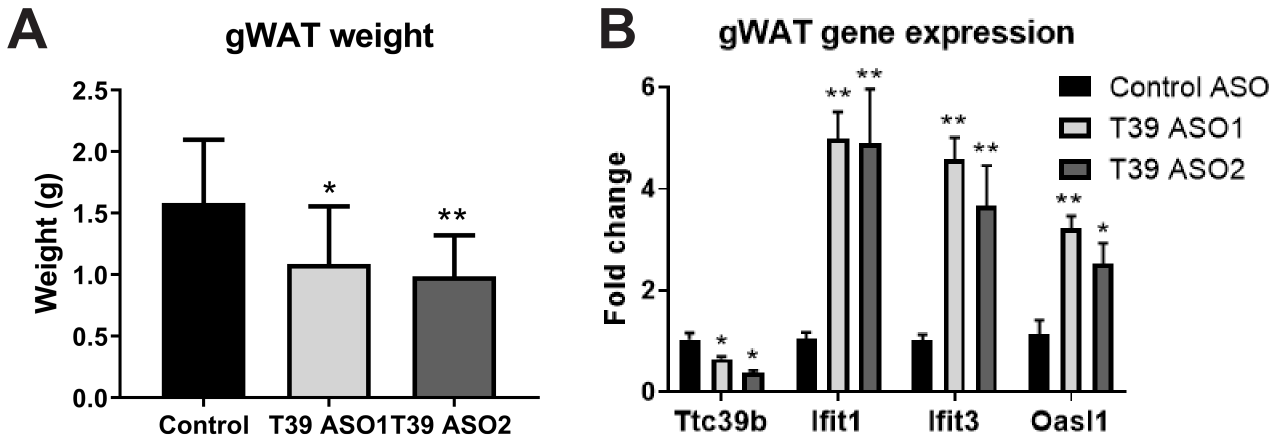

Supplemental figure 7: Mice treated with the control ASO, T39 ASO1, or T39 ASO2 and 4 weeks of HFSC diet. A) gWAT weight and B) gWAT gene expression measured by qRT-PCR normalized to *Actin* and *Cyclophilin* gene expression and expressed as fold change from control. Data represent mean ± SD (A) or SEM (B), n=10 mice/group, *$p<0.05$, **$p<0.01$.

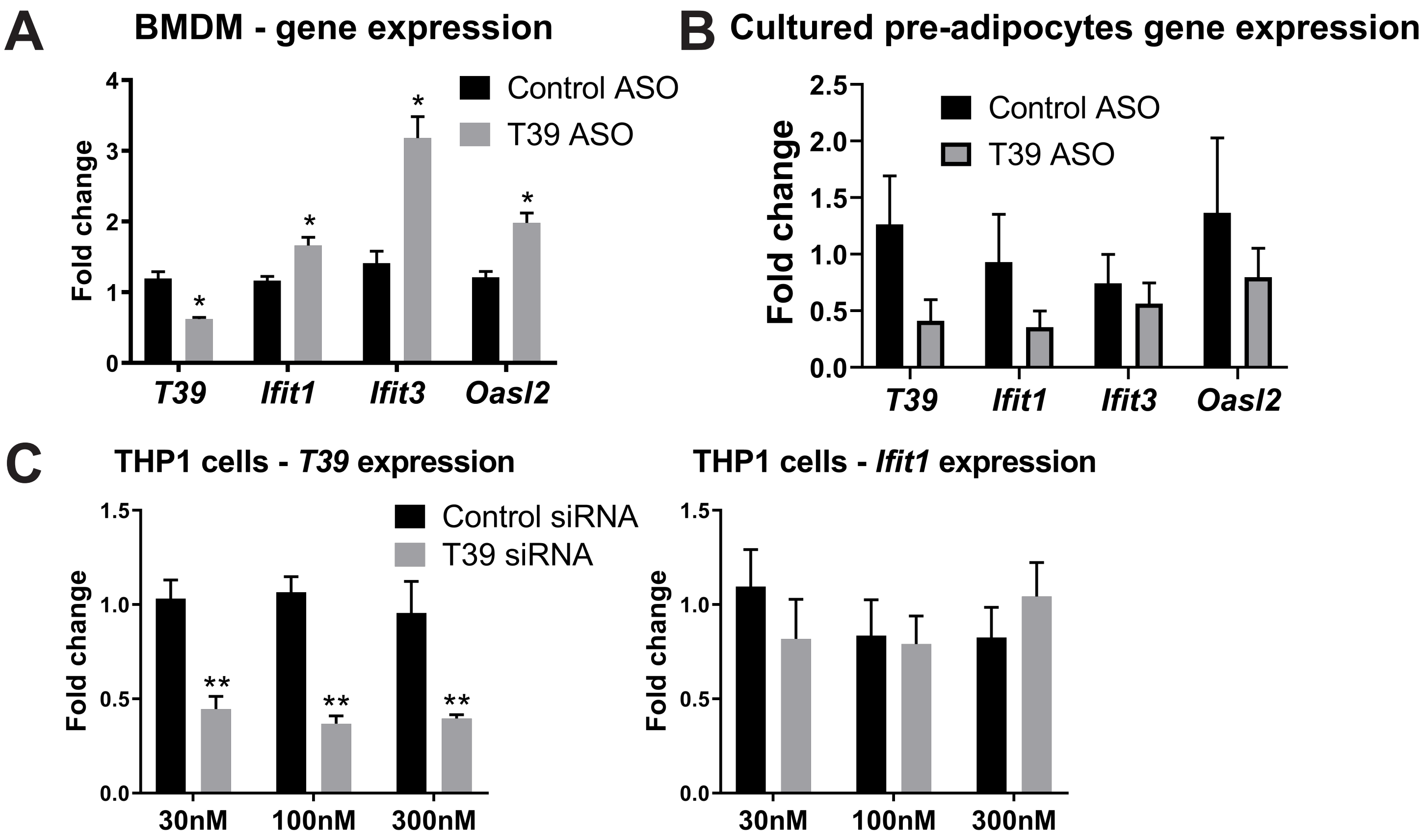

Supplemental figure 8: Gene expression measured by qRT-PCR normalized to *Actin* and *Cyclophilin* expression and expressed as fold change. A) BMDMs and B) pre-adipocytes isolated from C57Bl/6J mice and transfected with either control or T39 ASO and C) THP1 cells differentiated into macrophages with PMA and trasfected with either control or T39 siRNA. Data represents mean ± SEM, n=3-6/group, * $p<0.05$, ** $p<0.01$.
